# Supplementary material for: Structure of the native supercoiled flagellar hook as a universal joint
Source: Nat Commun. 2019 Nov 22;10:5295. doi: 10.1038/s41467-019-13252-9 (PMC6874566; doi:10.1038/s41467-019-13252-9)
Supplement: Supplementary file 1 — Supplementary Information [file 41467_2019_13252_MOESM1_ESM.pdf]

Supplementary Information

Structure of the native supercoiled flagellar hook as a universal joint

Kato et al.

Supplementary Information

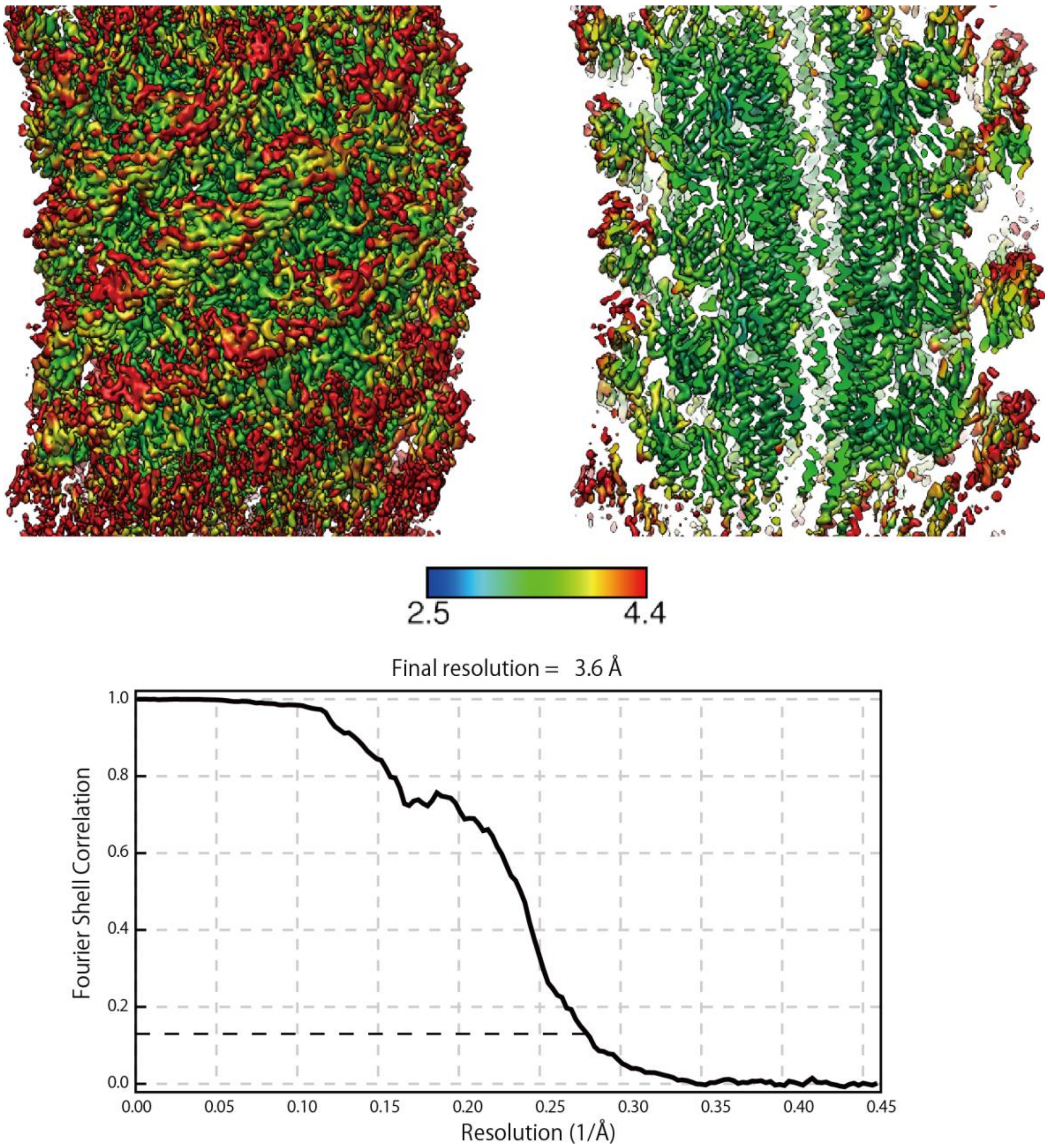

Supplementary Figure 1 Local and overall resolution of the native supercoiled hook structure. Colour maps of local resolution in the side view (upper left) and its cross-section (upper right). The blue to red gradient represents high to low resolution. The FSC curve of the reconstruction is shown in the lower panel.

Supplementary Information

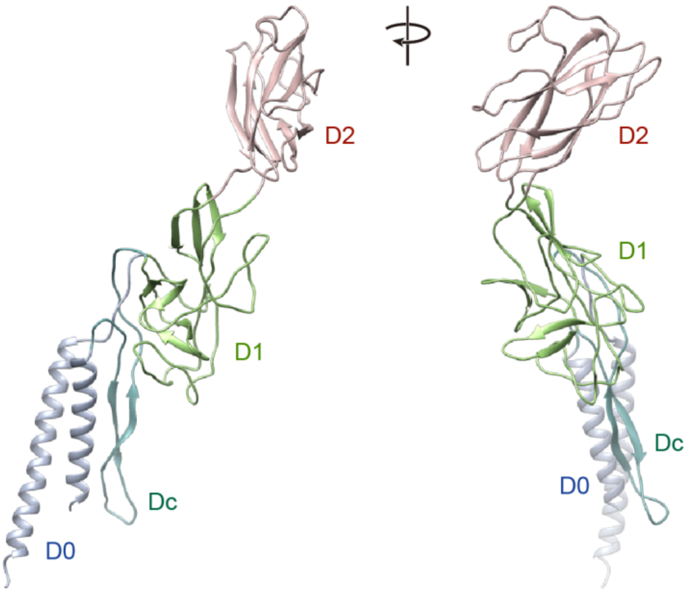

FlgE : SFSQAVSGLNAAATNLDVIGNNIANSATYGFKSGTASFADMFAGSKVGLGVKVAGITQDF (1–60; D0: 1–25, Dc: 26–60)

FlgE : TDGTTTNTGRGLDVAISQNGFFRLVDSNGSVFYSRNGQFKLDENRNLVNMQGMQLTGYPA (61–120; Dc: 61–71, D1: 72–120)

FlgE : TGTPPTIQQGANPAPITIPNTLMAAKSTTTASMQINLNSTDPVPSKTPFSVSDADSYNKK (121–180)

FlgE : GTVTVYDSQGNAHDMNVYFVKTKDNEWAVYTHDSSDPAATAPTTASTTLKFNENGILESG (181–240; D2)

FlgE : GTVNITTGTINGATAATFSLSFLNSMQQNTGANNIVATNQNGYKPGDLVSYQINNDGTVV (241–300)

FlgE : GNYSNEQEQVLGQIVLANFANNEGLASQGDNVWAATQASGVALLGTAGSGNFGKLTNGAL (301–360; D1)

FlgE : EASNVDLSKELVNMIVAQRNYQSNAQTIKTQDQILNTLVNLR (361–402; D0)

Supplementary Figure 2 Sequence of FlgE and regions of four domains, D0, Dc, D1 and D2. D0 (pale blue): Ser 1 – Asn 25, Gly 358 – Arg 402; Dc (blue-green): Ser 26 – Gly 71; D1 (light green): Leu 72 – Ala 145, Asp 287 – Asn 357; D2 (pink): Lys 146 – Gly 286.

#	1	2	3	4	5	6	7	8	9	10	11	12	13	14	15	16	17	18	19	20	21	22	23
1	0.00	1.23	1.57	2.23	2.96	3.69	3.98	3.58	2.73	1.87	1.28	1.11	1.20	1.56	2.19	2.98	3.58	3.98	3.65	2.86	1.90	1.35	1.38
2	1.23	0.00	1.32	2.02	2.69	3.47	3.78	3.43	2.60	1.86	1.36	1.22	1.19	1.33	1.90	2.75	3.34	3.76	3.50	2.74	1.84	1.41	1.41
3	1.57	1.32	0.00	1.48	2.09	2.89	3.20	2.94	2.25	1.76	1.48	1.46	1.41	1.04	1.39	2.17	2.75	3.17	2.98	2.34	1.66	1.50	1.66
4	2.23	2.02	1.48	0.00	1.41	2.04	2.36	2.15	1.79	1.83	2.02	2.20	2.12	1.58	1.06	1.43	1.91	2.34	2.21	1.83	1.70	1.97	2.24
5	2.96	2.69	2.09	1.41	0.00	1.45	1.68	1.66	1.71	2.17	2.58	2.87	2.82	2.19	1.44	1.19	1.28	1.69	1.68	1.67	2.02	2.52	2.88
6	3.69	3.47	2.89	2.04	1.45	0.00	1.26	1.38	1.93	2.62	3.29	3.62	3.61	2.99	2.18	1.56	1.10	1.32	1.38	1.85	2.56	3.19	3.60
7	3.98	3.78	3.20	2.36	1.68	1.26	0.00	1.27	1.95	2.86	3.51	3.88	3.89	3.29	2.47	1.79	1.19	1.08	1.26	1.88	2.72	3.42	3.85
8	3.58	3.43	2.94	2.15	1.66	1.38	1.27	0.00	1.55	2.38	3.12	3.50	3.56	3.02	2.29	1.78	1.35	1.32	1.11	1.54	2.28	3.00	3.48
9	2.73	2.60	2.25	1.79	1.71	1.93	1.95	1.55	0.00	1.63	2.22	2.60	2.72	2.33	1.81	1.73	1.78	1.96	1.59	1.26	1.49	2.11	2.61
10	1.87	1.86	1.76	1.83	2.17	2.62	2.86	2.38	1.63	0.00	1.48	1.82	1.95	1.85	1.79	2.22	2.58	2.86	2.49	1.81	1.16	1.40	1.85
11	1.28	1.36	1.48	2.02	2.58	3.29	3.51	3.12	2.22	1.48	0.00	1.20	1.38	1.51	1.89	2.62	3.15	3.50	3.14	2.38	1.42	1.06	1.37
12	1.11	1.22	1.46	2.20	2.87	3.62	3.88	3.50	2.60	1.82	1.20	0.00	1.18	1.49	2.07	2.90	3.48	3.86	3.55	2.76	1.78	1.22	1.23
13	1.20	1.19	1.41	2.12	2.82	3.61	3.89	3.56	2.72	1.95	1.38	1.18	0.00	1.44	2.02	2.87	3.48	3.88	3.60	2.86	1.95	1.46	1.40
14	1.56	1.33	1.04	1.58	2.19	2.99	3.29	3.02	2.33	1.85	1.51	1.49	1.44	0.00	1.45	2.22	2.85	3.27	3.08	2.41	1.75	1.52	1.66
15	2.19	1.90	1.39	1.06	1.44	2.18	2.47	2.29	1.81	1.79	1.89	2.07	2.02	1.45	0.00	1.50	2.03	2.44	2.30	1.85	1.65	1.85	2.14
16	2.98	2.75	2.17	1.43	1.19	1.56	1.79	1.78	1.73	2.22	2.62	2.90	2.87	2.22	1.50	0.00	1.39	1.75	1.75	1.71	2.08	2.55	2.86
17	3.58	3.34	2.75	1.91	1.28	1.10	1.19	1.35	1.78	2.58	3.15	3.48	3.48	2.85	2.03	1.39	0.00	1.21	1.31	1.78	2.42	3.05	3.47
18	3.98	3.76	3.17	2.34	1.69	1.32	1.08	1.32	1.96	2.86	3.50	3.86	3.88	3.27	2.44	1.75	1.21	0.00	1.28	1.89	2.71	3.39	3.82
19	3.65	3.50	2.98	2.21	1.68	1.38	1.26	1.11	1.59	2.49	3.14	3.55	3.60	3.08	2.30	1.75	1.31	1.28	0.00	1.51	2.34	3.06	3.50
20	2.86	2.74	2.34	1.83	1.67	1.85	1.88	1.54	1.26	1.81	2.38	2.76	2.86	2.41	1.85	1.71	1.78	1.89	1.51	0.00	1.69	2.27	2.73
21	1.90	1.84	1.66	1.70	2.02	2.56	2.72	2.28	1.49	1.16	1.42	1.78	1.95	1.75	1.65	2.08	2.42	2.71	2.34	1.69	0.00	1.35	1.81
22	1.35	1.41	1.50	1.97	2.52	3.19	3.42	3.00	2.11	1.40	1.06	1.22	1.46	1.52	1.85	2.55	3.05	3.39	3.06	2.27	1.35	0.00	1.39
23	1.38	1.41	1.66	2.24	2.88	3.60	3.85	3.48	2.61	1.85	1.37	1.23	1.40	1.66	2.14	2.86	3.47	3.82	3.50	2.73	1.81	1.39	0.00

Supplementary Figure 3 Pairwise root mean square deviations (RMSDs) of Cα atoms between 23 atomic models of FlgE subunits in the supercoiled hook. Large and small RMSDs are coloured red and blue, respectively.

a

#	D0	D1	D2
1	0.00	0.00	0.00
2	0.43	0.57	0.76
3	0.54	0.63	0.77
4	0.52	0.63	0.69
5	0.73	0.56	0.77
6	0.79	0.67	0.75
7	0.88	0.68	0.70
8	0.93	0.65	0.70
9	0.88	0.71	0.77
10	0.70	0.62	0.84
11	0.48	0.61	0.65

b

#	D0	D1	D2
1	0.43	0.57	0.76
2	0.00	0.00	0.00
3	0.40	0.61	0.74
4	0.44	0.63	0.65
5	0.63	0.57	0.77
6	0.75	0.72	0.74
7	0.88	0.70	0.77
8	0.96	0.67	0.71
9	0.90	0.68	0.77
10	0.74	0.67	0.84
11	0.53	0.62	0.76

c

#	D0	D1	D2
1	0.54	0.63	0.77
2	0.40	0.61	0.74
3	0.00	0.00	0.00
4	0.42	0.60	0.68
5	0.59	0.56	0.68
6	0.76	0.68	0.69
7	0.89	0.69	0.70
8	0.99	0.68	0.78
9	0.97	0.74	0.79
10	0.79	0.67	0.92
11	0.59	0.60	0.66

d

#	D0	D1	D2
1	0.52	0.63	0.69
2	0.44	0.63	0.65
3	0.42	0.60	0.68
4	0.00	0.00	0.00
5	0.53	0.52	0.68
6	0.63	0.63	0.63
7	0.78	0.60	0.70
8	0.83	0.59	0.73
9	0.83	0.62	0.73
10	0.68	0.62	0.82
11	0.55	0.59	0.62

e

#	D0	D1	D2
1	0.73	0.56	0.77
2	0.63	0.57	0.77
3	0.59	0.56	0.68
4	0.53	0.52	0.68
5	0.00	0.00	0.00
6	0.46	0.61	0.75
7	0.60	0.55	0.71
8	0.69	0.56	0.77
9	0.71	0.62	0.84
10	0.69	0.59	0.89
11	0.67	0.52	0.61

f

#	D0	D1	D2
1	0.79	0.67	0.75
2	0.75	0.72	0.74
3	0.76	0.68	0.69
4	0.63	0.63	0.63
5	0.46	0.61	0.75
6	0.00	0.00	0.00
7	0.43	0.61	0.78
8	0.49	0.61	0.69
9	0.57	0.62	0.78
10	0.60	0.57	0.76
11	0.71	0.62	0.70

g

#	D0	D1	D2
1	0.88	0.68	0.70
2	0.88	0.70	0.77
3	0.89	0.69	0.70
4	0.78	0.60	0.70
5	0.60	0.55	0.71
6	0.43	0.61	0.78
7	0.00	0.00	0.00
8	0.43	0.57	0.70
9	0.49	0.55	0.75
10	0.60	0.64	0.90
11	0.74	0.66	0.67

h

#	D0	D1	D2
1	0.93	0.65	0.70
2	0.96	0.67	0.71
3	0.99	0.68	0.78
4	0.83	0.59	0.73
5	0.69	0.56	0.77
6	0.49	0.61	0.69
7	0.43	0.57	0.70
8	0.00	0.00	0.00
9	0.49	0.61	0.86
10	0.53	0.59	0.81
11	0.77	0.64	0.72

i

#	D0	D1	D2
1	0.88	0.71	0.77
2	0.90	0.68	0.77
3	0.97	0.74	0.79
4	0.83	0.62	0.73
5	0.71	0.62	0.84
6	0.57	0.62	0.78
7	0.49	0.55	0.75
8	0.49	0.61	0.86
9	0.00	0.00	0.00
10	0.53	0.64	0.93
11	0.72	0.62	0.68

j

#	D0	D1	D2
1	0.70	0.62	0.84
2	0.74	0.67	0.84
3	0.79	0.67	0.92
4	0.68	0.62	0.82
5	0.69	0.59	0.89
6	0.60	0.57	0.76
7	0.60	0.64	0.90
8	0.53	0.59	0.81
9	0.53	0.64	0.93
10	0.00	0.00	0.00
11	0.56	0.61	0.80

k

#	D0	D1	D2
1	0.48	0.61	0.65
2	0.53	0.62	0.76
3	0.59	0.60	0.66
4	0.55	0.59	0.62
5	0.67	0.52	0.61
6	0.71	0.62	0.70
7	0.74	0.66	0.67
8	0.77	0.64	0.72
9	0.72	0.62	0.68
10	0.56	0.61	0.80
11	0.00	0.00	0.00

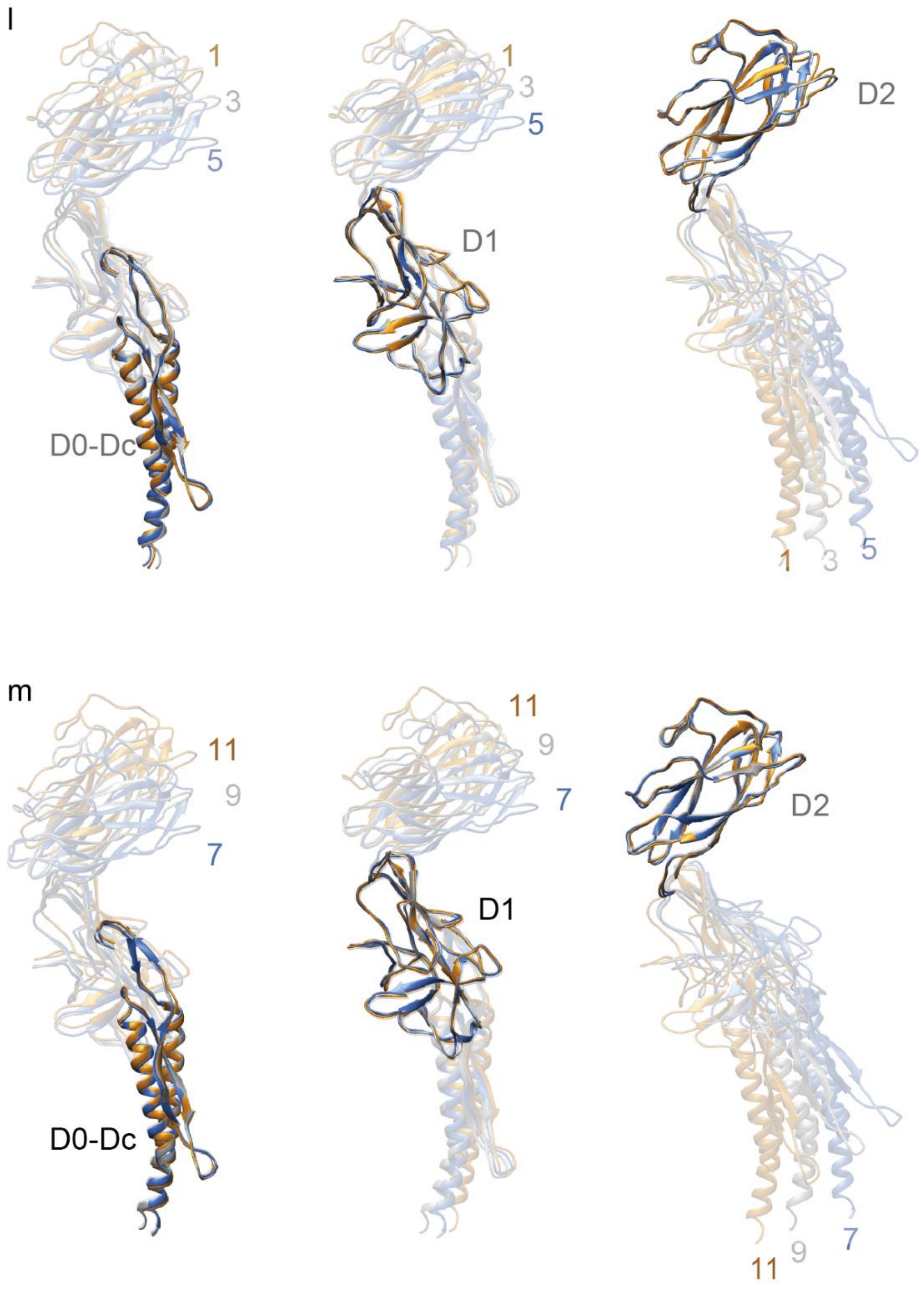

Supplementary Figure 4 Pairwise RMSDs of Cα atoms between 11 distinct conformations of FlgE subunit for each of three domains, D0-Dc, D1 and D2. Colours indicate the magnitude of RMSD with large and small RMSDs in red and blue, respectively. a – k, Each of 11 distinct conformations is used as a reference model for RMSD calculation. l and m, Superimposition of subunits 1, 3 and 5 in l and subunits 7, 9 and 11 in m, aligned with domains D0-Dc, D1 and D2, respectively.

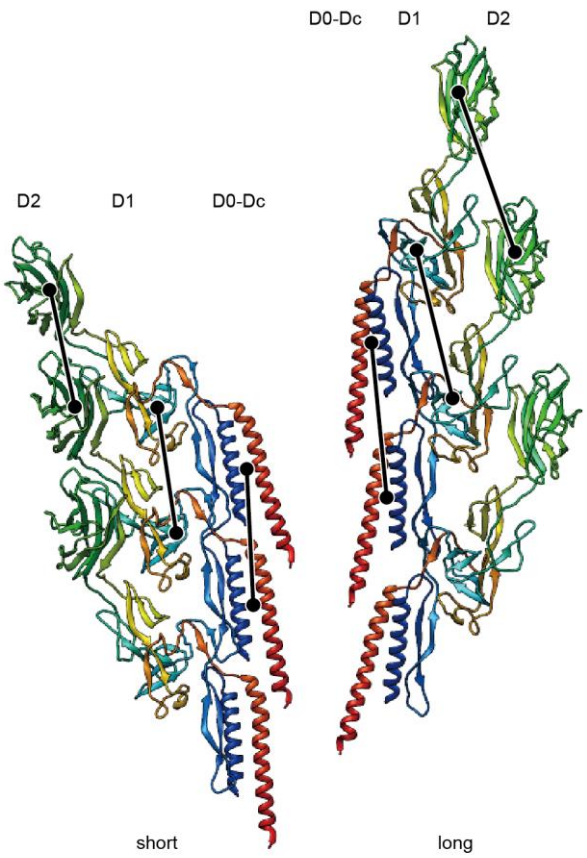

Domain	short	long	straight
D0-Dc	42.7	48.8	45.6
D1	40.6	50.4	45.6
D2	38.4	53.6	45.6

Supplementary Figure 5 Distances between neighboring subunits along the shortest and longest protofilaments and comparison with those of the straight hook structure. The distances are measured for each of three domains, D0-Dc, D1 and D2. The atomic model of the hook are coloured in rainbow from the N- to C-terminus.

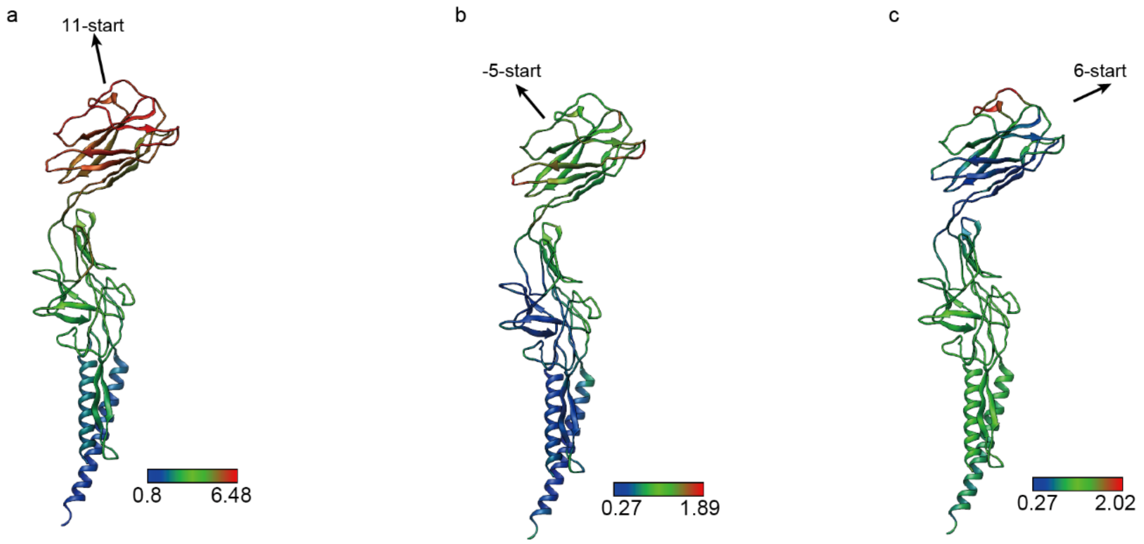

Supplementary Figure 6 Colour maps of standard deviations of distances between neighboring subunits in the 11-, -5 and 6-start helical directions measured over 11 distinct protofilament conformations. **a**, 11-start, **b**, -5-start, **c**, 6-start.

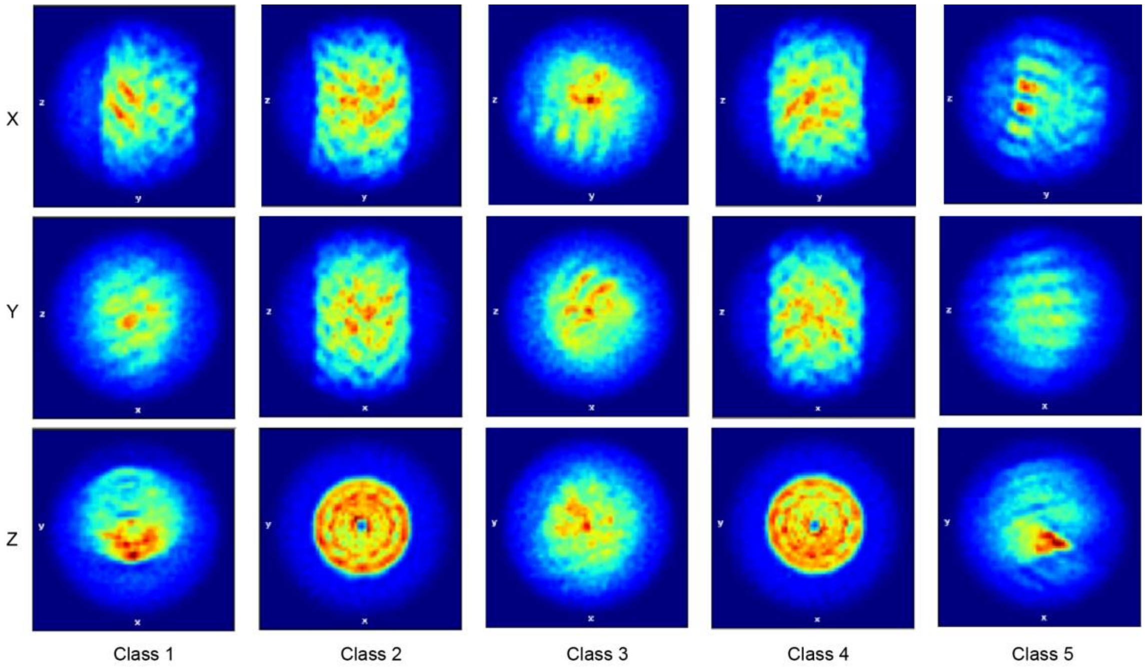

Supplementary Figure 7 Reprojections of the five 3D classes in ab-initio reconstruction. Classes 2 and 4 were selected for the homogeneous refinement.

Supplementary Information

Supplementary Table 1 Summary of cryoEM data collection, refinement and validation statistics

Parameter	value for	
Data collection and processing		
	Magnification	50,000
	Voltage (kV)	200
	Total exposure time (sec)	10
	Energy filter width (eV)	20
	Pixel size (Å/pixel)	1.097
	Total dose (e/Å^2)	50
	No. of frames	50
	Dose rate (e/Å^2/frame)	1.203
	No. of micrographs	1,702
	No. of initial particle images	1,029,196
	No. of final particle images	157,334
	Symmetry	C1
	Resolution (Å)	3.6
	FSC Threshold	0.143
	Map resolution range (Å)	2.5-4.4
Refinement		
	Model resolution cutoff (Å)	3.1
	Map sharpening B-factor	-125
Model composition		
	No. of protein residues	10,452
	No. of ligands	0
Validation		
	MolProbity score	1.92
	Clash score	9.41
	Poor rotamers	0.26
Ramachandran plot (%)		
	Favored	94.5
	Allowed	6.2
	Disallowed	0.07
