# Supplementary material for: Structure of the native supercoiled flagellar hook as a universal joint
Source: Nat Commun. 2019 Nov 22;10:5295. doi: 10.1038/s41467-019-13252-9 (PMC6874566; doi:10.1038/s41467-019-13252-9)
Supplement: Supplementary file 3 — Reporting Summary [file 41467_2019_13252_MOESM3_ESM.pdf]

nature research

Corresponding author(s): *Double-blind peer review submissions: write DBPR and your manuscript number here instead of author names.*

Last updated by author(s): *YYYY-MM-DD*

Reporting Summary

Nature Research wishes to improve the reproducibility of the work that we publish. This form provides structure for consistency and transparency in reporting. For further information on Nature Research policies, seeAuthors & Referees and theEditorial Policy Checklist .

Statistics

For all statistical analyses, confirm that the following items are present in the figure legend, table legend, main text, or Methods section.

n/a	Confirmed	
☒	☐	The exact sample size (n) for each experimental group/condition, given as a discrete number and unit of measurement
☒	☐	A statement on whether measurements were taken from distinct samples or whether the same sample was measured repeatedly
☒	☐	The statistical test(s) used AND whether they are one- or two-sided<br>*Only common tests should be described solely by name; describe more complex techniques in the Methods section.*
☒	☐	A description of all covariates tested
☒	☐	A description of any assumptions or corrections, such as tests of normality and adjustment for multiple comparisons
☒	☐	A full description of the statistical parameters including central tendency (e.g. means) or other basic estimates (e.g. regression coefficient) AND variation (e.g. standard deviation) or associated estimates of uncertainty (e.g. confidence intervals)
☒	☐	For null hypothesis testing, the test statistic (e.g. F, t, r) with confidence intervals, effect sizes, degrees of freedom and P value noted<br>*Give P values as exact values whenever suitable.*
☒	☐	For Bayesian analysis, information on the choice of priors and Markov chain Monte Carlo settings
☒	☐	For hierarchical and complex designs, identification of the appropriate level for tests and full reporting of outcomes
☒	☐	Estimates of effect sizes (e.g. Cohen's d, Pearson's r), indicating how they were calculated

Our web collection on statistics for biologists contains articles on many of the points above.

Software and code

Policy information about availability of computer code

Data collection	All images were collected by JADAS which is automated data accusation program produced by JEOL. The motion correction and CTF estimation were carried out in real-time by the Gwatch image processing pipeline software we developed. The link of Gwatch is https://github.com/FumiakiMakino/Gwatch/blob/master/Readme_jp.md.
Data analysis	MOTIONCOR2 was used for motion correction for movies. CTF estimation was carried out by GCTF (v1.06). REION (v3.0) was used for polyhook fragments detection, 2D classification and CTF refinement. cryoSPARC (v2.0) was used for Ab-initio reconstruction, homogeneous refinement and map sharpening. Atomic model building was performed by using COOT (v0.89). PHENIX (v1.15.2) was used for real-space refinement.

For manuscripts utilizing custom algorithms or software that are central to the research but not yet described in published literature, software must be made available to editors/reviewers. We strongly encourage code deposition in a community repository (e.g. GitHub). See the Nature Research guidelines for submitting code & software for further information.

Data

Policy information about availability of data

All manuscripts must include a data availability statement. This statement should provide the following information, where applicable:

- Accession codes, unique identifiers, or web links for publicly available datasets
- A list of figures that have associated raw data
- A description of any restrictions on data availability

The three-dimensional cryo-EM density map have been deposited into the Electron Microscopy Data Bank under accession numbers EMD-9952. The atomic model are deposited into the Protein Data Bank with accession number 6K9Q.

Field-specific reporting

Please select the one below that is the best fit for your research. If you are not sure, read the appropriate sections before making your selection.

- [x] Life sciences
- [ ] Behavioural & social sciences
- [ ] Ecological, evolutionary & environmental sciences

For a reference copy of the document with all sections, see nature.com/documents/nr-reporting-summary-flat.pdf

Life sciences study design

All studies must disclose on these points even when the disclosure is negative.

Sample size	No statistics methods were used to predetermine sample sizes. The 1702 movies were recorded in one beam time. From these movies, the 1,029,196 fragments were extracted and used for image processing. The 157,334 fragments were used for final reconstruction.
Data exclusions	After CTF estimation, the 315 movies which were bad focus or no hook movies were excluded. By 2D and 3D classification and selection, the 871,862 fragments were excluded from 1,029,196 fragments.
Replication	All attempts at replication were successful.
Randomization	Randomization is not relevant to this study, as the protein are not required to be allocated into experimental groups in protein structural studies, and no animals or human research participants are involved in this study.
Blinding	Blinding is not relevant to this study, as the protein are not required to be allocated into experimental groups in protein structural studies, and no animals or human research participants are involved in this study.

Reporting for specific materials, systems and methods

We require information from authors about some types of materials, experimental systems and methods used in many studies. Here, indicate whether each material, system or method listed is relevant to your study. If you are not sure if a list item applies to your research, read the appropriate section before selecting a response.

Materials & experimental systems

n/a	Involved in the study
☒	☐ Antibodies
☒	☐ Eukaryotic cell lines
☒	☐ Palaeontology
☒	☐ Animals and other organisms
☒	☐ Human research participants
☒	☐ Clinical data

Methods

n/a	Involved in the study
☒	☐ ChIP-seq
☒	☐ Flow cytometry
☒	☐ MRI-based neuroimaging
