# Supplementary material for: Structure of the native supercoiled flagellar hook as a universal joint
Source: Nat Commun. 2019 Nov 22;10:5295. doi: 10.1038/s41467-019-13252-9 (PMC6874566; doi:10.1038/s41467-019-13252-9)
Supplement: Supplementary file 4 — Description of Additional Supplementary Files [file 41467_2019_13252_MOESM4_ESM.docx]

**Description of Additional Supplementary Files**

File name: Supplementary Movie 1

Description: Structure of the native supercoiled hook of *Salmonella* flagellum

The 3D density map of the polyhook with a length of one helical pitch of the supercoil generated by extending the short segment map obtained by cryoEM image analysis followed by magnified images of the cryoEM map and fitted model.

File name: Supplementary Movie 2

Description: Structural changes of FlgE subunit during the universal motion of the hook

Structural changes of FlgE subunit shown in two orthogonal side views as the circumferential position of the protofilament changes as indicated in the upper left panel.

File name: Supplementary Movie 3

Description: Changes in the intersubunit interactions along the protofilament during the universal motion of the hook

Changes in the intersubunit interactions along the protofilament shown in two orthogonal side views as the circumferential position of the protofilament changes as indicated in the upper left panel.

File name: Supplementary Movie 4

Description: Universal joint motion of the native supercoiled hook transmitting motor torque generated by the flagellar basal body at the bottom (grey) to the filament extending towards an upper left direction (not displayed).
