# Supplementary material for: Structure of the native supercoiled flagellar hook as a universal joint
Source: Nat Commun. 2019 Nov 22;10:5295. doi: 10.1038/s41467-019-13252-9 (PMC6874566; doi:10.1038/s41467-019-13252-9)
Supplement: Supplementary file 2 — Peer Review [file 41467_2019_13252_MOESM2_ESM.pdf]

Reviewers' comments:

Reviewer #1 (Remarks to the Author):

In general, this is a strong paper that clearly deserves publication. The structure shows how a continuous series of conformations (11-states) explains the supercoiling of the flagellar hook, while current models for the flagellar filament are based upon 2-state models. The paper is therefore of much more general interest than simply to those studying bacterial motility, as it also demonstrates the new powers of cryo-EM to reconstruct at near-atomic resolution structures where symmetry does not need to be applied. I had a few technical concerns that need to be addressed by the authors.

It is stated in Extended Data Figure 1 that the resolution is 3.07 Å and in the paper this is rounded to 3.1 Å. Given that some people in the cryo-EM field describe resolution to a hundredth of an Å, it suggests that this precision is meaningful and significant. But if one actually looks at the figure shown, the first crossing of the FSC curve with the 0.143 threshold occurs at slightly worse than 1/(3.2 Å), as indicated by the arrow in my attached figure. More troubling, the curve never goes to 0.0, and plateaus to a value near 0.1 out to the Nyquist frequency. If this offset were to be subtracted from the curve, the stated resolution would be closer to 4.0 Å.

Extended Data Table 1 has some problems. It is stated that the voltage was 300 kV. If this was the case, they need to explain in the Methods how they were able to get a 200 keV microscope to operate at 300 keV. More likely it is a mistake. "Does rate" should be "Dose rate". And no units are given for "Total exposure time".

The paper needs to be carefully edited by someone who speaks English as a native language, as it is filled with typos and language that is uninterpretable. For example (lines 79-81): "Since the passes of the protofilaments are nearly parallel to the tubular axis of the hook, the protofilament length varies from one to the other." I assume that what is meant is "paths". Or line 85: "we built on the map" should be "we built into the map". Or line 112: "are achieved also by well-designed intersubunit interactions." Who designed them? What do they mean here? On line 179: "This difference in the mechanical property" should be "This difference in the mechanical properties". Or lines 199-200: "is now within the reach" which should be "is now within reach". This sentence (lines 186-190) is not atypical: "The corresponding gap in the Salmonella hook is much smaller in the compressed protofilaments (Extended Data Fig. 7), suggesting that the insertion of the FlgG specific 18-residues forms the L-stretch just as that of Campylobacter hook to fill the gap to prevent protofilament compression in the hook made of the insertion mutant of FlgE."

Reviewer #2 (Remarks to the Author):

The manuscript from Takayuki et al. presents the first cryo-EM structure of the native supercoiled flagellar hook at 3.1 Angstrom using a mutant fliK of Salmonella, which can be as long as 1um. This hook was proposed by the same group as the universal joint. With this resolution and the supercoiled conformation, the author can model all the 11 pf and also additional residues that is not modelled in their previous crystallography work. With this model, the authors can discuss about the difference between the shortest and longest units and see the interaction with the adjacent units for the universal join mechanism.

In my opinion, the results presented in the manuscript can attract broad interest and suitable for publication in Nature Communications. However, the manuscript, in particular, the figures needs a good modification in order to make the paper easy to read for the audience of Nat Comms. The manuscript is probably benefited a lot from a good cartoon to demonstrate what the readers are looking at, especially if they are not from the bacterial flagellar field.

Comments and suggestions

The 3 domains of FlgE were mentioned and discussed throughout the paper but none of this info is labelled in any main figures.

As a colour-blind person, it is extremely difficult more me to look at Figure 2a to distinguish any colour there.

In the text, it says "The superpositions of corresponding domains show almost no changes in their conformations as mentioned above (Extended Data Fig. 4). The extended data figure 4 is an RMSD table, which is informative but is terrible to convey the message. I would suggest having a superposition of each separate domain from PF 1,3,5 or 7,9,11 in Figure 2 to illustrate this point.

Figure 3a is very hard to understand if you are not coming from the bacterial flagellar field. A good schematic cartoon would help the reader to understand what is presented here in the neighbouring unit. For general readers, sudden presentation of -5 start, 11-start and 6-start will confuse them since the information they got from the figure before is the 11-protofilament.

In the text, there is a discussion about which patch of residues are interacting with which (Constant interactions are seen between residues Leu 101 – Glu 103 of subunit 0 with Ala 320 – Asn 321 and Gln 337 – Ser 339 of subunit 5 (Fig. 3d), but the interactions between Ser 87 – Asn 88 of subunit 6 and Gly 348 – Gly 350 of subunit 0 are present only in the compressed form). However, in Figure 3d, none of these residues are indicated in the figure.

There should be a figure to compare with flgE with flgE in straight hook conformation from Campylobacter jejuni. Only a part of this is presented in Extended Data Figure 7 but a full flgE should be compared.

For image Processing
What is the resolution attained during the refinement in cryoSPARC before & after CTF refinement in Relion 3.0?
Also, what is the 3 classes during classification look like? This info should be added in supplementary materials.

Minor Comments:
Why is the sampled incubated overnight at room temperature for prepare the native supercoiled hook?

Responses to the Reviewers' comments

To Reviewer #1:

In general, this is a strong paper that clearly deserves publication. The structure shows how a continuous series of conformations (11-states) explains the supercoiling of the flagellar hook, while current models for the flagellar filament are based upon 2-state models. The paper is therefore of much more general interest than simply to those studying bacterial motility, as it also demonstrates the new powers of cryo-EM to reconstruct at near-atomic resolution structures where symmetry does not need to be applied. I had a few technical concerns that need to be addressed by the authors.

Thank you very much for your recognition of the impact and strength of our study. We appreciate your technical concerns and made revisions in response to them as described below.

It is stated in Extended Data Figure 1 that the resolution is 3.07 Å and in the paper this is rounded to 3.1 Å. Given that some people in the cryo-EM field describe resolution to a hundredth of an Å, it suggests that this precision is meaningful and significant. But if one actually looks at the figure shown, the first crossing of the FSC curve with the 0.143 threshold occurs at slightly worse than 1/(3.2 Å), as indicated by the arrow in my attached figure. More troubling, the curve never goes to 0.0, and plateaus to a value near 0.1 out to the Nyquist frequency. If this offset were to be subtracted from the curve, the stated resolution would be closer to 4.0 Å.

The problem of the FSC curve not reaching 0.0 before the Nyquist frequency was due to redundant information by the 90% overlap of image segment boxes used for particle extraction. We therefore reprocessed images by editing the star file with RELION to avoid the use of redundant information in each half of the image data set for 3D image reconstruction. Now the FSC curve goes to 0.0, and the resolution is 3.6 Å at the FCS threshold of 0.143, as shown in Extended Data Fig. 1.

Extended Data Table 1 has some problems. It is stated that the voltage was 300 kV. If this was the case, they need to explain in the Methods how they were able to get a 200 keV microscope to operate at 300 keV. More likely it is a mistake. "Does rate" should be "Dose rate". And no units are given for "Total exposure time".

We apologize for the wrong and missing information in Extended Data Table 1. We revised it in a correct and complete form.

The paper needs to be carefully edited by someone who speaks English as a native language, as it is filled with typos and language that is uninterpretable. For example (lines 79-81): "Since the passes of the protofilaments are nearly parallel to the tubular axis of the hook, the protofilament length varies from one to the other." I assume that what is meant is "paths". Or line 85: "we built on the map" should be "we built into the map". Or line 112: "are achieved also by well-designed intersubunit interactions." Who designed them? What do they mean here? On line 179: "This difference in the mechanical property" should be "This difference in the mechanical properties". Or lines 199-200: "is now within the reach" which should be "is now within reach". This sentence (lines 186-190) is not atypical: "The corresponding gap in the Salmonella hook is much smaller in the compressed protofilaments (Extended Data Fig. 7), suggesting that the insertion
of the FlgG specific 18-residues forms the L-stretch just as that of Campylobacter hook to fill the gap to prevent protofilament compression in the hook made of the insertion mutant of FlgE."

Thank you for the list of our typographic mistakes. We made revisions according to them and carefully went through the entire manuscript to make correction in English.

To Reviewer #2:

The manuscript from Takayuki et al. presents the first cryo-EM structure of the native supercoiled flagellar hook at 3.1 Angstrom using a mutant fliK of Salmonella, which can be as long as 1um. This hook was proposed by the same group as the universal joint. With this resolution and the supercoiled conformation, the author can model all the 11 pf and also additional residues that is not modelled in their previous crystallography work. With this model, the authors can discuss about the difference between the shortest and longest units and see the interaction with the adjacent units for the universal join mechanism.

In my opinion, the results presented in the manuscript can attract broad interest and suitable for publication in Nature Communications. However, the manuscript, in particular, the figures needs a good modification in order to make the paper easy to read for the audience of Nat Comms. The

manuscript is probably benefited a lot from a good cartoon to demonstrate what the readers are looking at, especially if they are not from the bacterial flagellar field.

Thank you very much for your favorable comments on our study. We appreciate your comments and suggestions and made revisions in response to them as described below.

Comments and suggestions

The 3 domains of FlgE were mentioned and discussed throughout the paper but none of this info is labelled in any main figures.

Domains D0, D1 and D2 are now labeled in Fig. 1 and 3.

As a colour-blind person, it is extremely difficult more me to look at Figure 2a to distinguish any colour there.

We apologize for the colors in Fig. 2 that are difficult to recognize. We change them to blue to orange. We hope they are all right.

In the text, it says "The superpositions of corresponding domains show almost no changes in their conformations as mentioned above (Extended Data Fig. 4). The extended data figure 4 is an RMSD table, which is informative but is terrible to convey the message. I would suggest having a superposition of each separate domain from PF 1,3,5 or 7,9,11 in Figure 2 to illustrate this point.

Superpositions of FlgE with each separate domain for PF 1,3,5 and 7,9,11 are presented as panels l and m of Extended Data Fig. 4.

Figure 3a is very hard to understand if you are not coming from the bacterial flagellar field. A good schematic cartoon would help the reader to understand what is presented here in the neighbouring unit. For general readers, sudden presentation of -5 start, 11-start and 6-start will confuse them since the information they got from the figure before is the 11-protofilament.

We added a figure as Fig. 3a showing a short hook segment with the subunit array on the surface by rainbow colored D2 domains, in which four neighboring subunits are labeled with numbers and the three major helical lines of -5, 11-start and 6-start are indicated as a guide for readers.

In the text, there is a discussion about which patch of residues are interacting with which (Constant interactions are seen between residues Leu 101 – Glu 103 of subunit 0 with Ala 320 – Asn 321 and Gln 337 – Ser 339 of subunit 5 (Fig. 3d), but the interactions between Ser 87 – Asn 88 of subunit 6 and Gly 348 – Gly 350 of subunit 0 are present only in the compressed form). However, in Figure 3d, none of these residues are indicated in the figure.

We put residue labels as many as possible in new Fig. 4, which was part of original Fig. 3.

There should be a figure to compare with flgE with flgE in straight hook conformation from Campylobacter jejuni. Only a part of this is presented in Extended Data Figure 7 but a full flgE should be compared.

We moved Extended Data Figure 7 to the main text as Figure 6 and added a figure panel comparing *Salmonella* FlgE with *Campylobacter* FlgE as Figure 6a.

For image Processing

What is the resolution attained during the refinement in cryoSPARC before & after CTF refinement in Relion 3.0?

The resolution attained during the refinement in cryoSPARC before and after CTF refinement in RELION 3.0 was 3.3 Å and 3.1 Å, respectively. However, as mentioned in our response to Reviewer #1, in order to solve a problem of the FSC curve not reaching zero, we reprocessed image data by avoiding the use of redundant information caused by the 90% overlap of image segment boxes used

for particle extraction, and the resolution is now 3.6 Å after CTF refinement in RELION 3.0. We described this in Methods and updated the FSC curve in Extended Data Fig. 1.
Also, what is the 3 classes during classification look like? This info should be added in supplementary materials.
We used 5 classes in redong the image processing and used 2 classes. They are shown in Extended Data Fig. 7.
Minor Comments:
Why is the sampled incubated overnight at room temperature for prepare the native supercoiled hook?
The hook is supercoiled at room temperature but becomes straight at temperatures below 4ºC. This is how we carry out cryoEM helical image analysis of the straight hook.
